# Porcine Hemagglutinating Encephalomyelitis Virus Co-Opts Multivesicular-Derived Exosomes for Transmission

**DOI:** 10.1128/mbio.03054-22

**Published:** 2022-12-21

**Authors:** Zi Li, Shaoqian Mu, Yihan Tian, Junchao Shi, Yungang Lan, Jiyu Guan, Kui Zhao, Feng Gao, Wenqi He

**Affiliations:** a Key Laboratory for Zoonosis Research of the Ministry of Education, College of Veterinary Medicine, Jilin University, Changchun, China; National Institutes of Health

**Keywords:** betacoronavirus, porcine hemagglutinating encephalomyelitis virus, exosome, transmission, innate response

## Abstract

Porcine hemagglutinating encephalomyelitis virus (PHEV) is a member of the family *Coronaviridae*, genus *Betacoronavirus*, and subgenus *Embecovirus* that causes neurological disorders, vomiting and wasting disease (VWD), or influenza-like illness (ILI) in pigs. Exosomes regulate nearby or distant cells as a means of intercellular communication; however, whether they are involved in the transmission of viral reference materials during PHEV infection is unknown. Here, we collected exosomes derived from PHEV-infected neural cells (PHEV-exos) and validated their morphological, structural, and content characteristics. High-resolution mass spectrometry indicated that PHEV-exos carry a variety of cargoes, including host innate immunity sensors and viral ingredients. Furthermore, transwell analysis revealed that viral ingredients, such as proteins and RNA fragments, could be encapsulated in the exosomes of multivesicular bodies (MVBs) to nonpermissive microglia. Inhibition of exosome secretion could suppress PHEV infection. Therefore, we concluded that the mode of infectious transmission of PHEV is likely through a mixture of virus-modified exosomes and virions and that exosomal export acts as a host strategy to induce an innate response in replicating nonpermissive bystander cells free of immune system recognition.

## OBSERVATION

Betacoronaviruses (β-CoVs) are positive-strand enveloped RNA viruses that infect humans and domestic and wild animals, resulting in complex manifestations of multiorgan disease (1). Transcription and replication of their full-length negative-strand RNA occur in a membrane system consisting of convoluted membranes and double-membrane vesicles (DMVs), which are both derived from the rough endoplasmic reticulum (ER). The newly synthesized viral genomic RNA, coated with N proteins and decorated with the structural proteins S, E, and M, buds into the ER-Golgi intermediate compartment (ERGIC) (2). After assembly, the progeny virions’ egress from cells is an important determinant of viral infectivity and pathogenicity. Betacoronaviruses, including severe acute respiratory syndrome coronavirus 2 (SARS-CoV-2) and mouse hepatitis virus (MHV), exploit the Arf-like small GTPase Arl8b-dependent lysosomal pathway or secretory vesicles for release into the extracellular environment (3–5). While the life cycle of coronaviruses is well understood, little is known regarding how progeny virions communicate between infected and uninfected cells. Most recently, diverse effects of exosomes of endocytic origin on coronavirus disease 2019 (COVID-19) have been suggested, from viral transmission to therapeutic application (6). Exosomes, which are 30 to 120 nm, originate from multivesicular bodies (MVBs) of virtually all cells and are released by the fusion of MVBs with the cell membrane (7). Once secreted, exosomes are distributed into the extracellular fluid and deliver bioactive proteins, lipids, and nucleic acids to neighboring and distantly located target cells (7). The structure and biogenesis of exosomes resemble those of viruses, in particular enveloped RNA viruses, strongly indicating that exosomes and viruses cross paths in biological functions (8). A growing body of evidence indicates that exosomes participate in a reenactment of *War and Peace* between RNA viruses and their hosts. During HIV infection, exosomes facilitate infection and immune evasion by spreading cellular components (such as the viral receptors CCR5 and CXCR4) to increase the pool of susceptible cells or by absorbing antiviral antibodies (9–11). In contrast, exosomes can also combat infections. For example, interferon-stimulated exosomal hACE2 inhibits SARS-CoV-2 replication by competitively blocking virus entry and releasing exosomes containing SARS-CoV-2 S protein, along with critical microRNAs, into circulation to suppress innate immunity (12–14). In addition, exosomes directly package viral components, such as proteins and nucleic acid fragments, to regulate the cellular microenvironment (15, 16).

**FIG 1:**
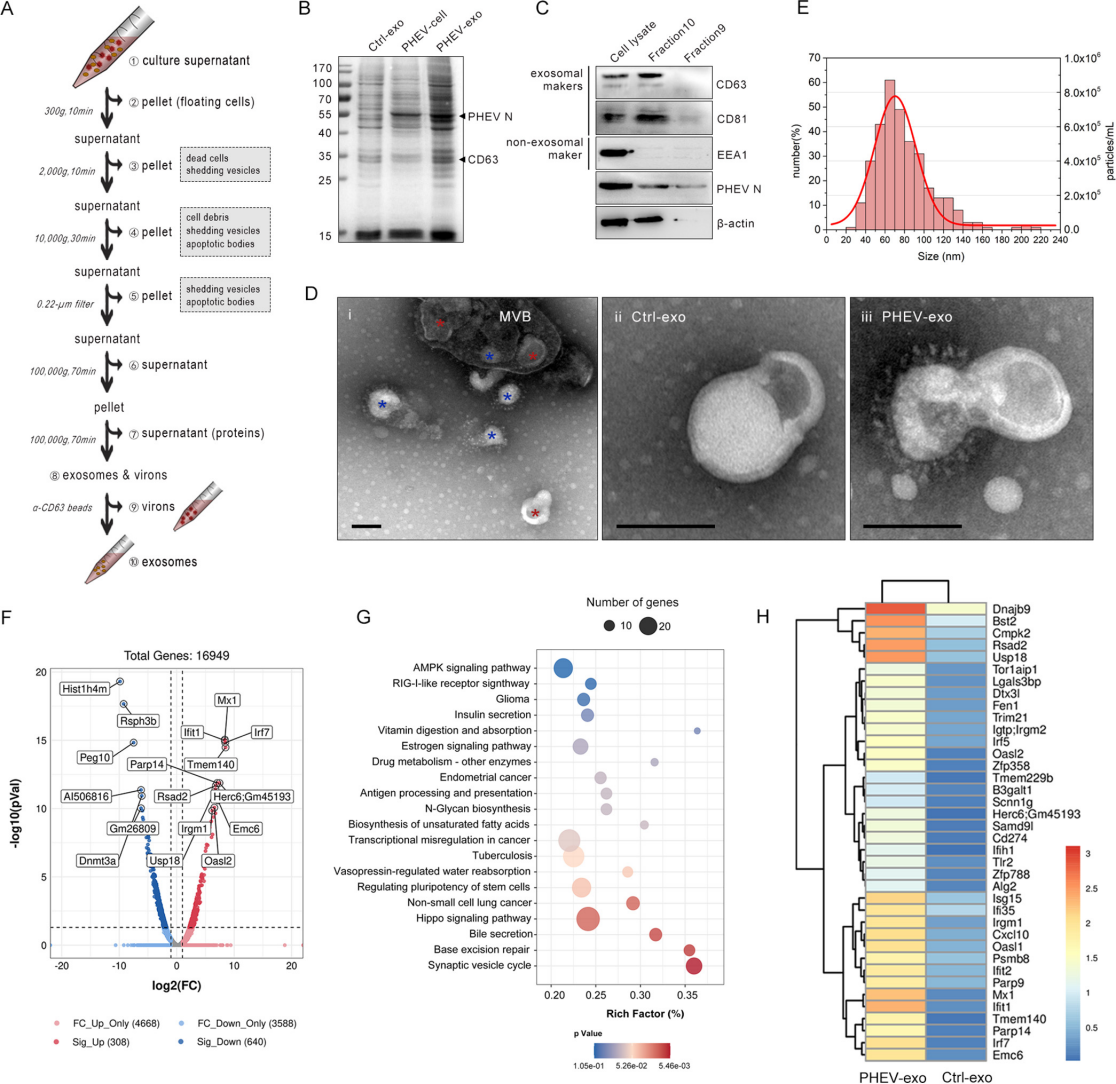
Isolation, identification, and bioinformatics analysis of exosomes. (A) Magnetic bead-based isolation of exosomes. Exosomes from N2a cells infected with PHEV (multiplicity of infection [MOI], 50) for 48 h were isolated by ultracentrifugation and then purified using CD63 immunomagnetic beads. (B) SDS-PAGE analysis of exosomes (Ctrl-exo and PHEV-exo) and PHEV-infected N2a cells (PHEV-cell). (C) Total cell lysate, viral materials (fraction 9), and isolated exosomes (fraction 10) were collected, and then the exosomal markers CD63 and CD81, early endosomal marker EEA1, and PHEV N protein were measured by Western blot analysis. β-actin was used as a loading control. (D) Transmission electron microscopy (TEM) analysis of fractions 8 (i) and 10 (ii and iii). Scale bar, 100 nm. Red asterisks, exosomes. Blue asterisks, virions. (E) The particle size of the exosomes secreted by PHEV-infected N2a cells was measured by nanoparticle tracking analysis. (F) Transcriptome sequencing (RNA-seq) of purified PHEV-exos and Ctrl-exos was performed using an Illumina HiSeq 4000 system. Of 16,949 genes detected, the 17 genes with the most significant changes are marked on a volcano plot. (G) KEGG enrichment of pathways. Mean ± SD values are shown, with two-tailed unpaired *t* tests. (H) Heatmap of the differentially expressed genes in relation to immune response. The data are representative of at least three independent experiments.

Given that coronaviruses are highly similar to exosomes in size and biogenesis, it has been assumed that enveloped virions use vesicles of this secretory pathway for intercellular transmission (11). Here, we began investigating cell-to-cell communication during the course of coronaviral infection using the β-CoV porcine hemagglutinating encephalomyelitis virus (PHEV), an important causative agent of acute neurological disease in piglets and mice (17, 18). Mouse neuroblastoma N2a cells were first infected with PHEV stocks for 24 h, at which point the cells were not lytic or apoptotic (see Fig. S1A and B in the supplemental material). Exosomes were then isolated from the culture supernatant by differential ultracentrifugation and CD63 immunomagnetic purification (fractions 1 to 10), as illustrated in Fig. 1A. Immunoblotting of the CD63-purified pellet (fraction 10) showed one band at a low density, consistent with CD63-membrane association, whereas the upper band was at the density expected for viral materials (Fig. 1B). PHEV N protein and exosomal markers (CD63 and CD81) were detected in the isolated exosomes by Western blotting (Fig. 1C). Transmission electron microscopy (TEM) showed that the intracellular PHEV infectious particles were trapped within MVBs in fraction 8, along with exosomes (Fig. 1D, panel i). In fraction 10, exosomes derived from control-infected N2a cells (Ctrl-exos) had a typical cup-shaped structure (Fig. 1D, panel ii), while exosomal outer membrane-associated PHEV particles (PHEV-exos) were frequently observed in the PHEV-infected group (Fig. 1D, panel iii). Nanoparticle tracking analysis (NTA) revealed that the diameter of PHEV-exos was approximately 50 to 100 nm (Fig. 1E). These structurally intact exosomes derived from PHEV-infected N2a cells (i.e., PHEV-exos) were decorated with spike-like structures, whose morphology was similar to that of the S glycoprotein structure on the surface of PHEV particles and was readily detected by immunoelectron microscopy (Fig. S1C). Further characterization by flow cytometry confirmed the presence of the two types of exosomes in the PHEV-exo fraction (Fig. S1D). In addition, a projection of electron microscopy illustrated that these closed MVBs were filled with significant numbers of virions, dense bodies, and other structures that we dubbed “virus-decorated exosomes” (Fig. S2). These findings indicate that membrane-associated PHEV particles are present within MVBs, together with decorated exosomes. The structure and biogenesis of exosomes resemble those of enveloped RNA viruses, in particular coronaviruses in assembly, strongly suggesting that exosomes and coronaviruses cross paths in biological functions.

10.1128/mbio.03054-22.1 FIG S1 Surface characteristics of PHEV-modified exosomes. (A) Hoechst 33342/propidium iodide (PI) double staining and trypan blue staining. N2a cells were infected with PHEV for 24 h or treated with the apoptosis inducer Staurosporine (STS; 1.0 μM) for 12 h. Bars, 100 μm. (B) Annexin V/7-aminoactinomycin D (7-AAD) for apoptosis staining in flow cytometry. Cells were treated as indicated in panel A. (C) Exosome fractions from culture supernatant of PHEV-uninfected (i) or PHEV-infected cells (ii). Red asterisk, exosomes. Immunogold labeling of PHEV-modified exosomes (iii) or PHEV particles (iv) with antimouse PHEV-S polyclonal antibody and a 16-nm colloidal gold-conjugated goat antimouse IgG. Red asterisks, exosomes. Blue asterisks, virions. Yellow asterisks, PHEV-modified exosomes decorated with a spike structure of ~10 nm. Scale bars, 200 nm. (D) Flow cytometry analysis of CD63 Dynabead-captured exosomes isolated from PHEV-uninfected (Ctrl-exo) or PHEV-infected N2a cells (PHEV-exo). The isolated exosomes were stained for the typical exosomal marker CD81. Download FIG S1, TIF file, 12.8 MB. Copyright © 2022 Li et al. 2022 Li et al. https://creativecommons.org/licenses/by/4.0/ This content is distributed under the terms of the Creative Commons Attribution 4.0 International license.

10.1128/mbio.03054-22.2 FIG S2 PHEV virions and virus-modified exosomes accumulate within the MVBs of N2a cells. (A) TEM images of N2a cells infected with PHEV at an MOI of 50 for 24 h. PHEV virions packaged in MVBs in the cytoplasm or attached to the cell membrane surface are labeled with red arrows. MVB, multivesicular bodies. M, mitochondria. Yellow asterisks, virus-decorated exosomes. Red arrows, virions. Scale bar, 1.0 μm. (B) TEM images of N2a cells infected with PHEV at an MOI of 50 for 48 h. MVBs carrying assembled virions are released extracellularly through membrane fusion or rupture of the cell membrane (dotted box). Download FIG S2, TIF file, 1.9 MB. Copyright © 2022 Li et al. 2022 Li et al. https://creativecommons.org/licenses/by/4.0/ This content is distributed under the terms of the Creative Commons Attribution 4.0 International license.

**FIG 2:**
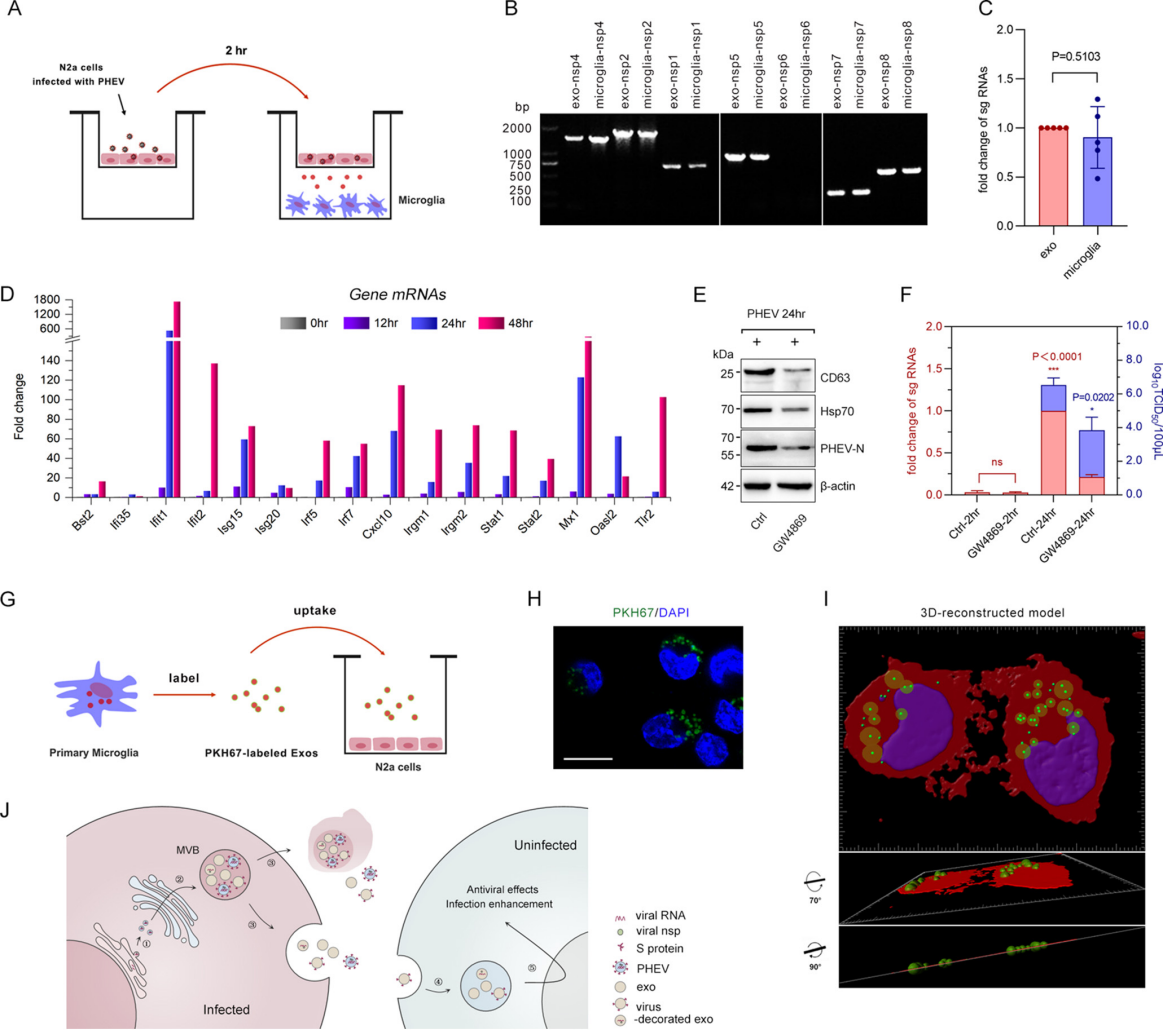
Exosome packaging viral components and immunity sensors are transferred to microglia. (A) N2a cells infected with PHEV (MOI, 50) for 2 h were cocultured with primary microglia for 24 h in a transwell plate (membrane pore size, 0.4 μm). (B) PHEV RNA fragments (nsp1, nsp2, and nsp4 to nsp8) were detected in microglial cytoplasm (microglia) and exosomes isolated from the supernatant of the coculture system (exo). (C) RT-qPCR showed no significant differences in PHEV sgRNA levels between the two groups. (D) Expression of innate immune genes (indicated in Fig. 1H) in microglial cytoplasm was determined using RT-qPCR and normalized to β-actin. Data are presented as the means (*n* = 3). (E and F) The N2a cells in the upper transwell plate were treated with GW4689 (10 μM) for 24 h and then incubated with PHEV for 2 h. After removing the culture supernatant, the treated N2a cells were transferred to a coculture system that was preseeded with microglia in the lower plate, as indicated in panel A. After 2 or 24 h of cocultivation, the levels of PHEV N protein and sgRNAs in the N2a cells were detected by Western blot (E) and RT-qPCR (F), respectively. The virus titer in the supernatant of the coculture system was tested using the 50% tissue culture infective dose (TCID_50_) method. Mean ± SD values are shown, with two-tailed unpaired *t* tests; ***, *P* < 0.0001; *, *P* = 0.0202. (G) Exosomes isolated from primary microglia were labeled with PKH67 and added to N2a cell cultures. (H) Green fluorescence represents the uptake of PKH67-labeled exosomes by N2a cells, as shown by confocal laser scanning microscope tomography. Scale bar, 50 μm. (I) Quantification of 3D-reconstructed z-stacks was performed using the Imaris program. Green, PKH67-labeled exosomes. Red, cytomembrane. Purple, cell nucleus. (J) Exosomes mediate the cell-to-cell transmission of virus infectious and antiviral activity. Exosomes and PHEV particles are simultaneously released by infected N2a cells and share pathways for biogenesis at MVBs. MVBs contain both virions and virus-decorated exosomes and are released from the cell after fusion of the MVB with the plasma membrane or after plasmatorrhexis. Exosomes packaging viral genome fragments and viral (glyco)proteins are endocytosed by uninfected immune cells, microglia, to trigger antiviral activity and facilitate infection.

Next, we sought to investigate the global cargo of exosomes. Transcriptome sequencing (RNA-seq) of purified PHEV-exos and Ctrl-exos was performed using an Illumina HiSeq 4000 system. KEGG pathway enrichment analysis mining of 308 upregulated genes (fold change [FC], ≥2.88; *P* < 0.05) that were significantly more abundant in PHEV-exos showed that immune responses, inflammatory responses, and ubiquitin-mediated proteolysis were universally modulated (Fig. 1F and G; Text S1). Innate immune effectors, pattern recognition receptors (PRRs), and interferon-stimulated genes (ISGs) (such as ISG15, OASL1, and OASL2) related to antiviral immunity were captured by heatmap analysis (Fig. 1H and Table S1). Considering the close relationships between exosome biogenesis and virus assembly, we used a transwell assay to monitor the transmission of PHEV RNA replication intermediates. As expected, viral nonstructural proteins (nsps) and subgenomic RNAs (sgRNAs) were readily detected in nonpermissive microglia, after 24 h of cocultivation with PHEV-permissive N2a cells (Fig. 2A to C and Table S2). The activation of microglial innate immunity was verified by reverse transcriptase quantitative PCR (RT-qPCR) (Fig. 2D). Among the most elevated genes were interferon-induced proteins with tetratricopeptide repeats 1 (Ifit1/p56/ISG56), which confer antiviral defense downstream of type I interferon through disruption of the host translation initiation machinery, and myxovirus resistance 1 (Mx1), whose activity is required for exerting antiviral effects. This suggests that the release of PHEV-exos in culture could incorporate and transfer antiviral signatures via a virus particle-independent pathway. Prior addition of the neutral sphingomyelinase inhibitor GW4869 to the transwell blocked exosome production and delivery of viral components from N2a cells into microglia (Fig. 2E and F). Furthermore, we labeled microglia-derived exosomes with the fluorescent dye PKH67 and then seeded them into a cocultivation system. After 24 h, the N2a cells exhibited efficient uptake of the microglia-secreted exosomes and transported them via the MVB pathway, as indicated by 3D reconstructed confocal z-stacks employing Imaris software (Fig. 2G to I). Together, these data revealed that PHEV-permissive cells transfer viral ingredients or antiviral activity and can communicate with nearby or distant cells by egressing a mixture of exosomes and virions.

10.1128/mbio.03054-22.3 TABLE S1 Upregulated genes associated with immune responses. Download Table S1, DOCX file, 0.01 MB. Copyright © 2022 Li et al. 2022 Li et al. https://creativecommons.org/licenses/by/4.0/ This content is distributed under the terms of the Creative Commons Attribution 4.0 International license.

10.1128/mbio.03054-22.4 TABLE S2 Sequences of primers used for the RT-qPCR assay. Download Table S2, DOCX file, 0.01 MB. Copyright © 2022 Li et al. 2022 Li et al. https://creativecommons.org/licenses/by/4.0/ This content is distributed under the terms of the Creative Commons Attribution 4.0 International license.

10.1128/mbio.03054-22.5 TEXT S1 Raw data from RNA-seq. Download Text S1, XLSX file, 0.7 MB. Copyright © 2022 Li et al. 2022 Li et al. https://creativecommons.org/licenses/by/4.0/ This content is distributed under the terms of the Creative Commons Attribution 4.0 International license.

Host circulating exosomes, mesenchymal stem cell (MSC)-derived exosomes, and other extracellular vesicles carrying viral and host components play significant roles in both facilitating and suppressing coronavirus infection (19). In this scenario, our studies demonstrate that during PHEV infection of neural cells, multiple viral components and host factors are encapsulated in MVB-derived exosomes for cell-to-cell communication in the extracellular environment (Fig. 2J). Interestingly, in PHEV infection, various diverse exosomes are released, so that at one extreme, there are exosomes decorated with virus-like structures, such as spikes. As we pointed out, the fact that both exosomes and PHEV virions use the cellular MVB-associated vesiculation machinery explains their striking similarities. These virus-modified exosomes have a physiological role in the host-pathogen standoff, and they can be recognized as a pathogen-associated molecular pattern (PAMP) carrier that mediates the transfer of immunostimulatory cargo from infected cells to uninfected cells. However, it is not yet known whether virus-modified exosomes containing viral proteins and fragments of the viral genome actually cause outbreaks and epidemics. For example, we demonstrated that PHEV nsp1, nsp2, nsp4, nsp5, nsp7, and nsp8 can be carried by exosomes for transmission, but nsp6 is an exception. Given that coronavirus nsp6 has membrane proliferation ability and induces perinuclear vesicles localized around the microtubule organizing center (20), we hypothesized that exosomes and perinuclear vesicles may have different biofilm formation mechanisms, which will require more research efforts in the future. Notably, the exosome biogenesis/release inhibitor GW4869 resulted in a significant reduction in PHEV RNA *in vitro*, but is this enough to show a therapeutic effect on animal disease? This is one of the dilemmas of current research. Although the specific roles of exosomes in antiviral defenses and inflammation during β-CoV infection remain unclear, the exosomal transfer process in relation to transmission (6), infection, diagnosis, treatment, therapeutics, drug delivery, and vaccines represents a novel intervention for the treatment of diseases such as COVID-19. There are still many problems to be solved for disease treatment, such as modification of natural exosomes to enhance their ability to cross multiple biological barriers and enhance tissue-targeted capabilities. Future research on exosomes should focus on the development of engineering methods and delivery systems. As functional nanomaterials, exosomes have safer and more effective therapeutic properties for difficult diseases and offer intriguing tools as potential vaccines due to their ability to deliver a wide range of antigens and immunomodulatory properties.
