# Supplementary figures and images for: Porcine Hemagglutinating Encephalomyelitis Virus Co-Opts Multivesicular-Derived Exosomes for Transmission
Source: mBio. 2022 Dec 21;14(1):e03054-22. doi: 10.1128/mbio.03054-22 (PMC9973304; doi:10.1128/mbio.03054-22)

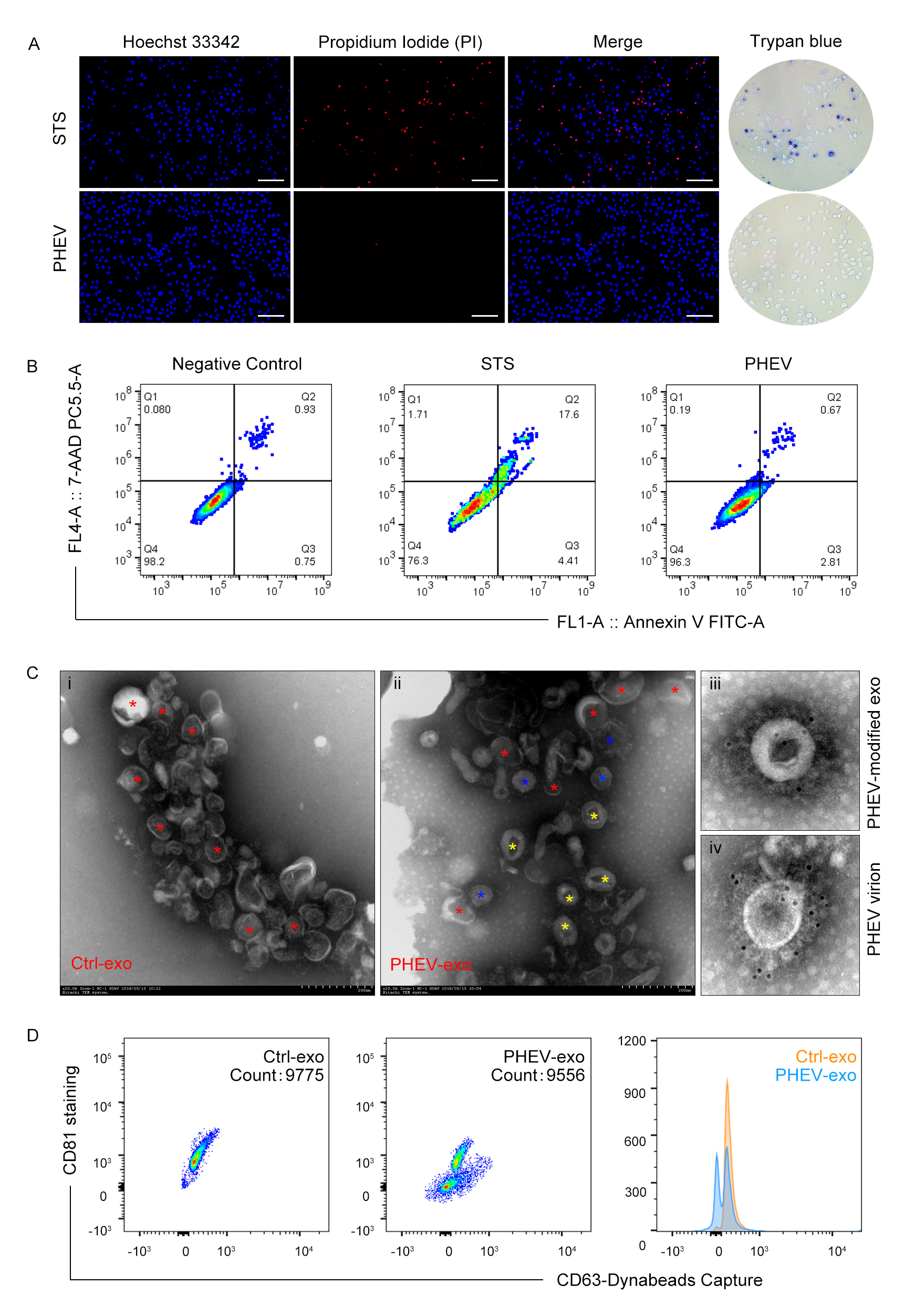

Supplement: FIG S1 [file mbio.03054-22-s0001.tif]

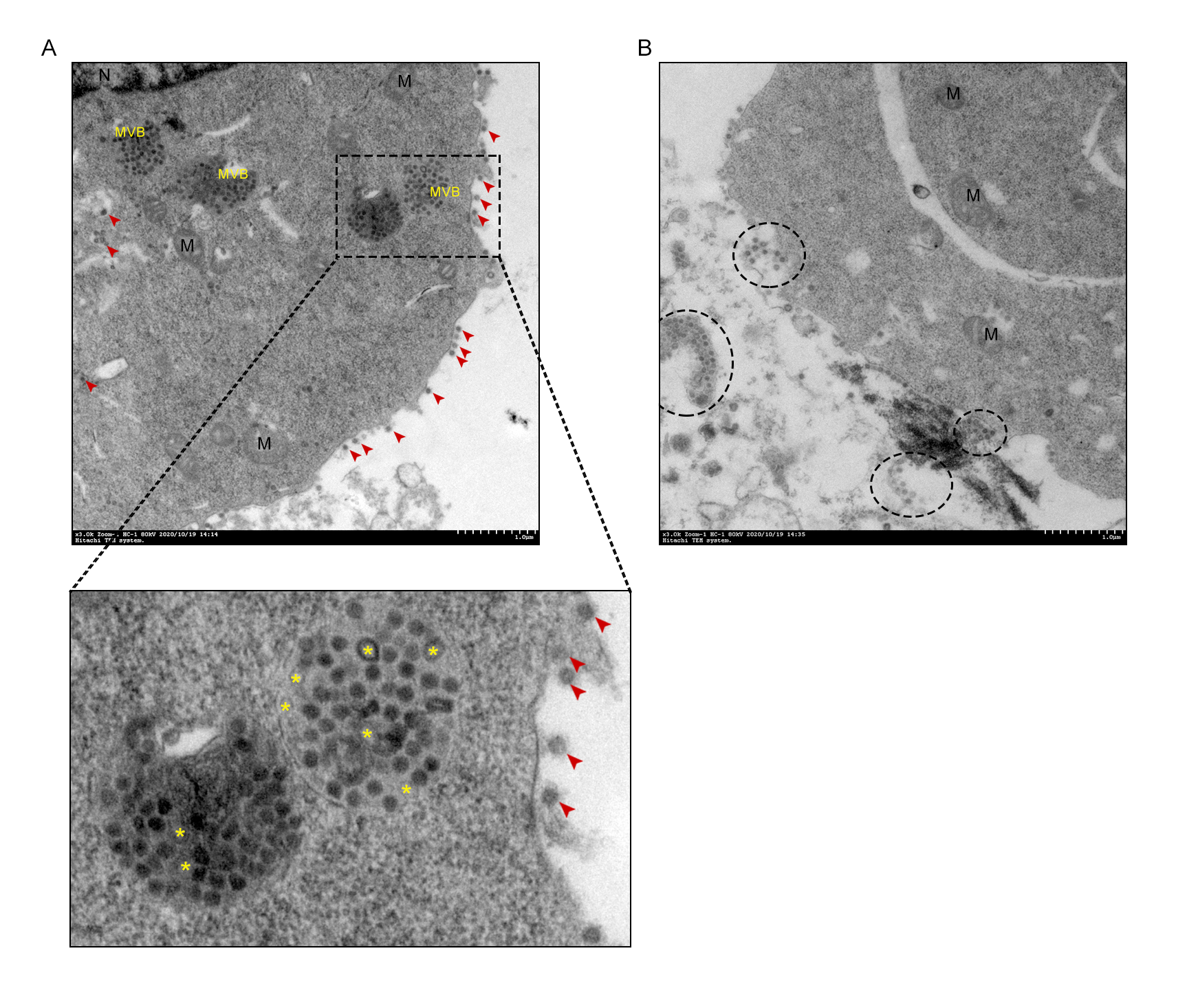

Supplement: FIG S2 [file mbio.03054-22-s0002.tif]
