# Supplementary material for: Porcine Hemagglutinating Encephalomyelitis Virus Co-Opts Multivesicular-Derived Exosomes for Transmission
Source: mBio. 2022 Dec 21;14(1):e03054-22. doi: 10.1128/mbio.03054-22 (PMC9973304; doi:10.1128/mbio.03054-22)
Supplement: TABLE S1 [file mbio.03054-22-s0003.docx]

**TABLE S1** Up-regulated genes assoicated with immune responses

| **Pathway** | **Genes** | **P value** |
| --- | --- | --- |
| RIG-I-like receptor signaling pathway | Cxcl10; Ddx58; Dhx58; Fadd; Ifih1; Irf7; Isg15; Mapk11; Tbkbp1; Tmem173; Trim25 | 0.10 |
| Toll-like receptor signaling pathway | Akt3; Cxcl10; Fadd; Irf5; Irf7; Map2k1; Mapk11; Mapk3; Myd88; Pik3r2; Stat1; Gm28177; Tlr2; Tlr4 | 0.35 |
| Jak-STAT signaling pathway | Akt3; Cbl; Ccnd3; Cntfr; Ghr; Grb2; Il13ra1; Jak2; Pik3r2; Pim1; Spred2; Spred3; Spry4; Stat1; Gm28177; Stat2 | 0.36 |
| NOD-like receptor signaling pathway | Birc2; Cxcl1; Mapk11; Mapk3; Naip2; Nod1; Pstpip1 | 0.62 |
| NF-kappa B signaling pathway | Birc2; Ddx58; Erc1; Myd88; Parp1; Pidd1; Tlr4; Trim25 | 0.79 |
| Ubiquitin mediated proteolysis | Birc2; Cbl; Cul7; Fancl; Fzr1; Herc2; Mdm2; Mid1; Rchy1; Rnf7; Siah1a; Trim32; Ube2c; Ube2e2; Ube2l6; Ube3b | 0.92 |
