# Supplementary material for: Porcine Hemagglutinating Encephalomyelitis Virus Co-Opts Multivesicular-Derived Exosomes for Transmission
Source: mBio. 2022 Dec 21;14(1):e03054-22. doi: 10.1128/mbio.03054-22 (PMC9973304; doi:10.1128/mbio.03054-22)
Supplement: TABLE S2 [file mbio.03054-22-s0004.docx]

**TABLE S2** Sequences of primers used for the RT-qRNA assay

| **Primer name** | **Primer sequence** | **Product (bp)** |
| --- | --- | --- |
| nsp1 | F：TCGAAGATCAACAAATACGGT | 732 |
|  | R：ACCACGAATATCCTCTAATTAAAGCAT |  |
| nsp2 | F：GTTAAACCGCTTCTCTATGTAGAC | 1815 |
|  | R：AGCACAAGGAACTCTCCATACT |  |
| nsp4 | F：GCAGTTTTTAGTTATTTTGTGT | 1488 |
|  | R：TTGCAAAAATGAAGTTGAC |  |
| nsp5 | F：TCAGGTATTGTGAAAATGG | 909 |
|  | R：TTGTAACTTGATACCAGC |  |
| nsp6 | F：TCAAAGCGCACTAGATT | 861 |
|  | R：TTGAAATTGAGATACTTCAATGA |  |
| nsp7 | F：TCAAAATTGACTGATGTCAAA | 267 |
|  | R：CTGCAAAACAGTATTGTCC |  |
| nsp8 | F：GCTTTACAGAGTGAATTTGT | 591 |
|  | R：TTGCAAAACGGTAGCAG |  |
